# Supplementary material for: Quorum-sensing synthase mutations re-calibrate autoinducer concentrations in clinical isolates of Pseudomonas aeruginosa to enhance pathogenesis
Source: Nat Commun. 2023 Dec 2;14:7986. doi: 10.1038/s41467-023-43702-4 (PMC10693556; doi:10.1038/s41467-023-43702-4)
Supplement: Supplementary file 1 — Supplementary Information [file 41467_2023_43702_MOESM1_ESM.pdf]

Supplementary Information

Quorum-sensing synthase mutations re-calibrate autoinducer concentrations in clinical isolates of *Pseudomonas aeruginosa* to enhance pathogenesis

Kayla A. Simanek[1], Megan L. Schumacher[1], Caleb P. Mallery[1], Stella Shen[2], Lingyun Li[3], and Jon E. Paczkowski[1,2]

[1]Department of Biomedical Sciences, University at Albany, School of Public Health, Albany, New York, 12201, United States

[2]Division of Genetics, Wadsworth Center, New York State Department of Health, Albany, New York, 12208, United States

[3]Division of Environmental Health Sciences, Wadsworth Center, New York State Department of Health, Albany, New York, 12208, United States

Corresponding author: Jon Paczkowski; e-mail address: jon.paczkowski@health.ny.gov

Contents:

I. Supplementary Figures

II. Supplementary Tables

III. Supplementary References

I. Supplementary Figures

a

b

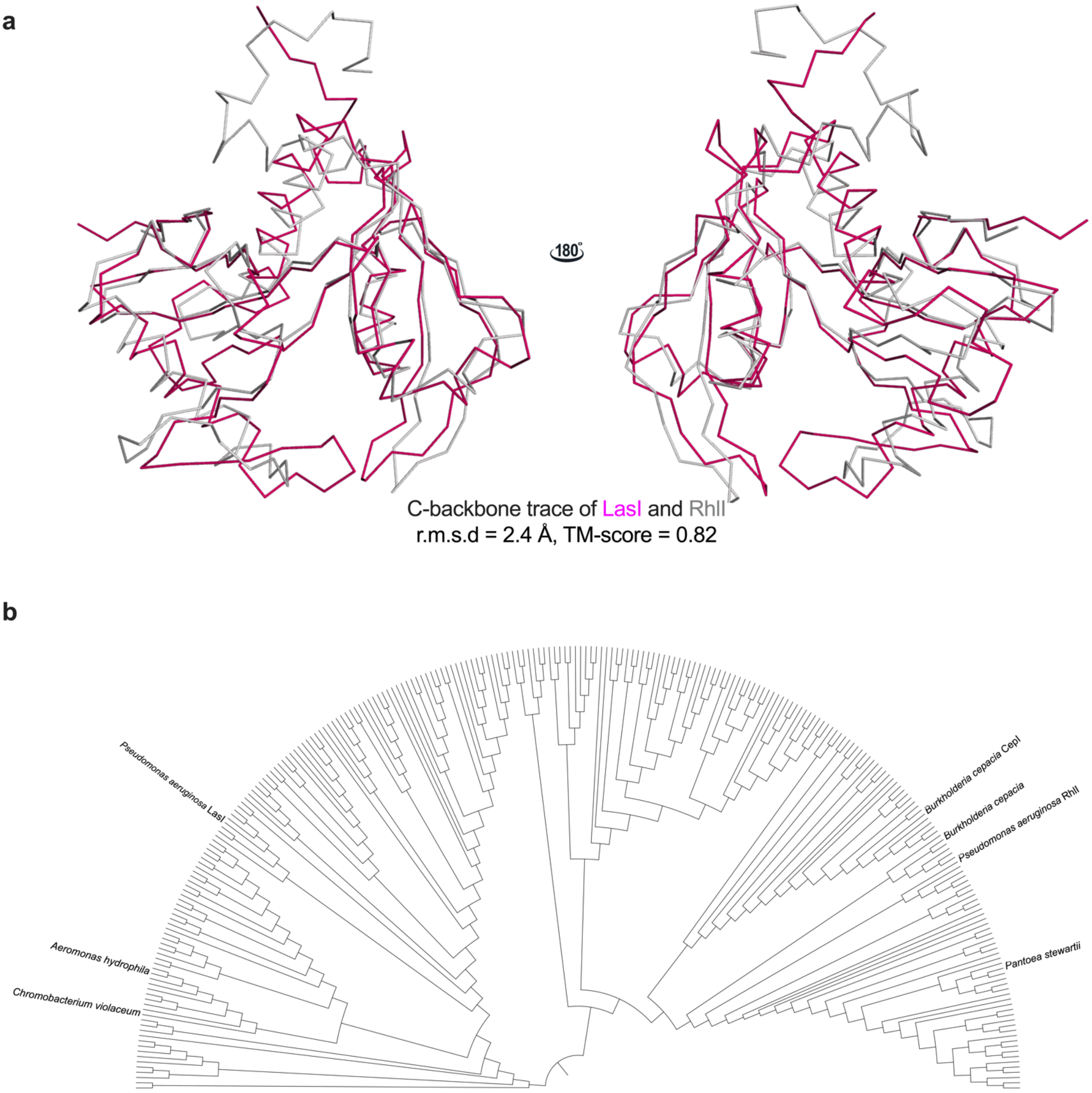

Figure S1: Structural comparison of AHL-synthases. (a) Structural overlay of the C-backbone of LasI (magenta) and RhlI (gray). The r.m.s.d. (root-mean-squared deviation) and TM-score were determined using the DockRMSD docking pose distance calculation[1]. Protein pairs with a TM-score >0.5 are considered to be nearly the same fold. (b) Phylogenetic tree of RhlI orthologous sequences, highlighting the species *Chromobacterium violaceum*, *Aeromonas hydrophila*, *P. aeruginosa, Burkholderia cepacia*, and *Panotea stewartii*. The phylogenetic reconstruction was based on the alignment of 264 orthologous protein sequences obtained from OrthoDB v11[2].

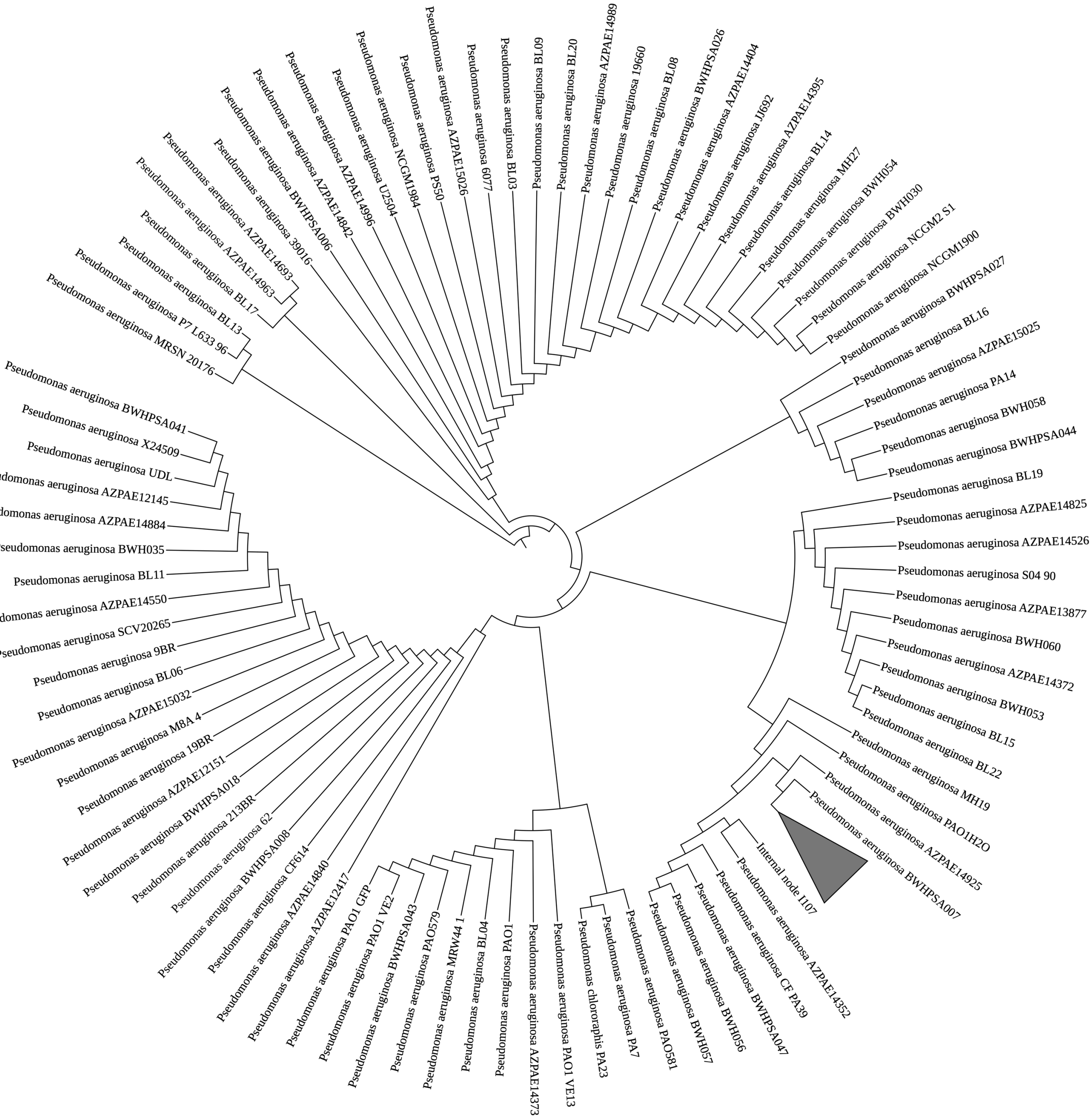

Figure S2. Distribution of RhlI variants in sequenced isolates of *P. aeruginosa*. Phylogenetic reconstruction of RhlI orthologues protein sequences among *P. aeruginosa* genomes (*n* = 295) publicly available in *Pseudomonas* Genome database[3]. Distribution of RhlI variants in isolates: G62S 32/295 (10.8 %), D83E 245/295 (83.1 %), P159S 1/295 (0.01 %). Collapsed clade indicates 204 nodes with a branch length of zero.

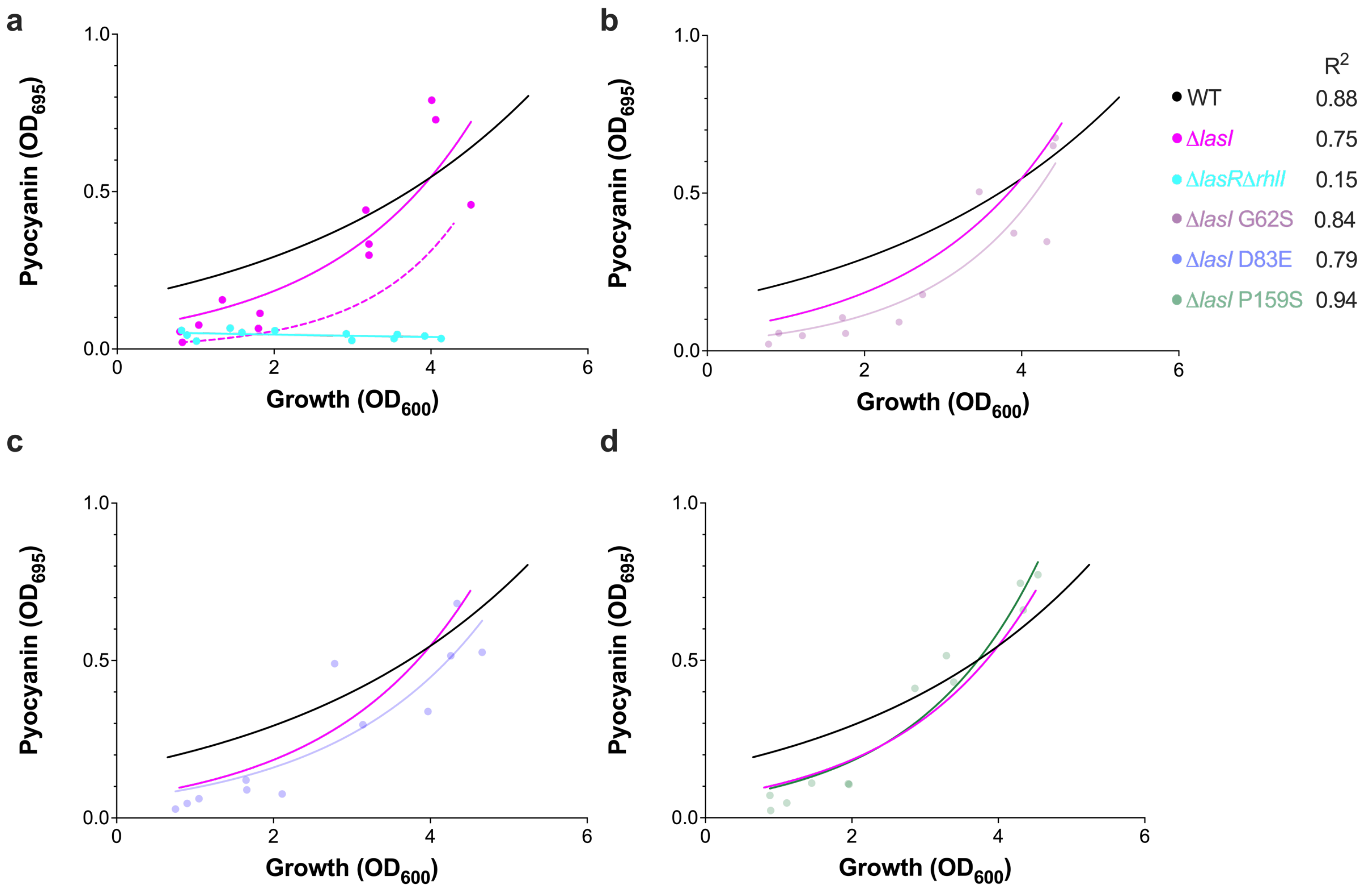

Figure S3: RhlI variants do not alter pyocyanin production in a Δ*lasI* background. (a) Summary graph of pyocyanin time course experiment for Δ*lasI* (magenta dots) and Δ*lasR*Δ*rhlI* (cyan) strains. Measurements were taken at four time points over a 24-hour period (approximately 5, 10, 18 and 24 hours). The Δ*lasR* data (dotted magenta line) from Figure 2 is shown for reference. Time course data is plotted for b) Δ*lasI* RhlI G62S (light purple); c) Δ*lasI* RhlI D83E (light blue); d) Δ*lasI* RhlI P159S (light green). One measurement was made per strain per time point. The experiment was performed in triplicate, resulting in the 12 total data points depicted per strain. Non-linear regression analyses were performed for every genotype and the best fits shown; best fit lines for WT (from Figure 2) (black) and Δ*lasI* (magenta) control strains were plotted and copied for reference in subsequent panels for the RhlI variant strains.

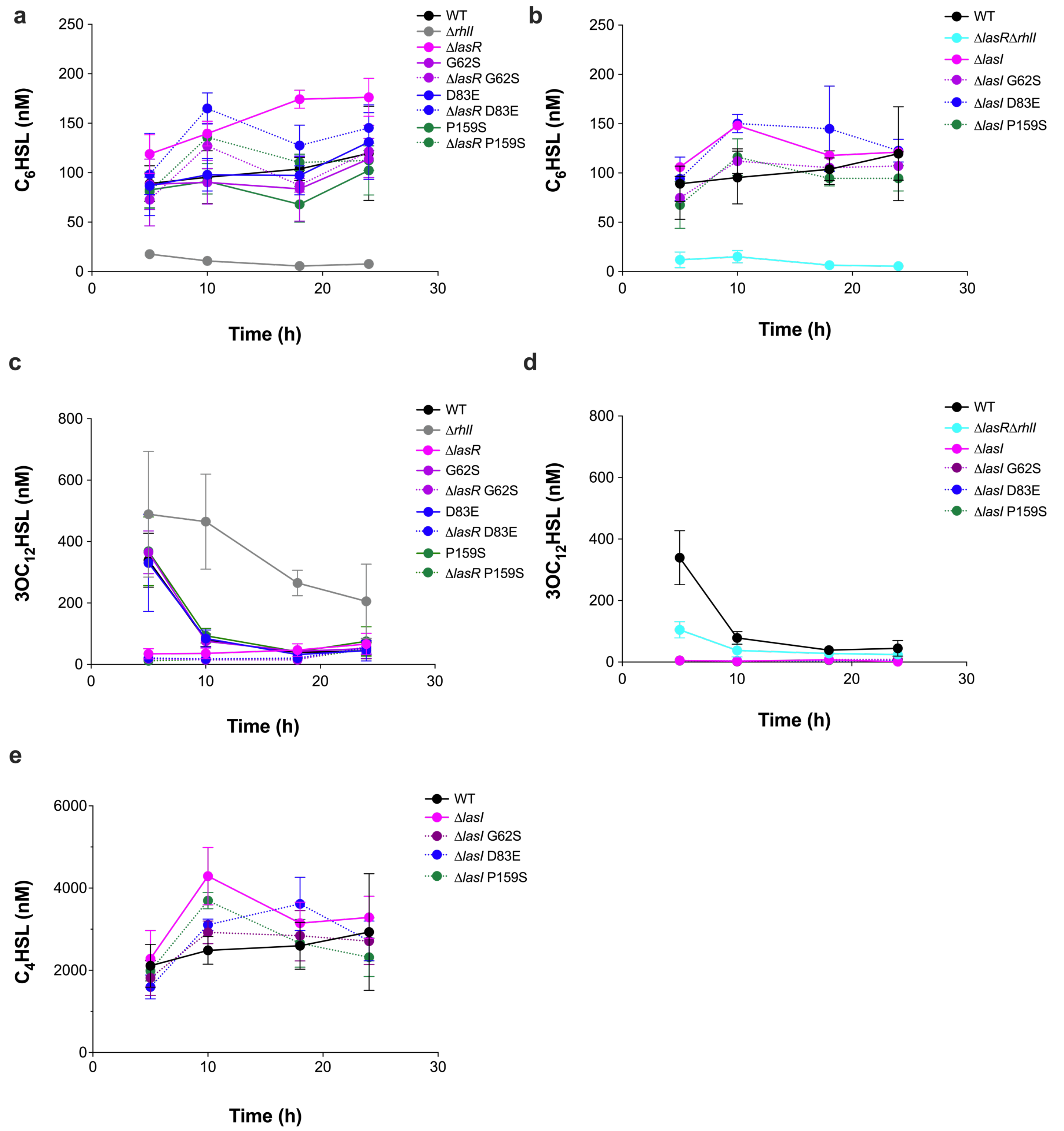

Figure S4: AHL levels produced in *P. aeruginosa* mutant strains measured by HRMS over time. Absolute concentrations of C_6HSL and $3OC_{12}$HSL synthesized by RhlI variants in a Δ*lasR* background (a,c) and a Δ*lasI* background (b,d), respectively. (e) C_4HSL concentrations synthesized by RhlI variants in a Δ*lasI* background. Bars represent the mean of three biological replicates. Error bars represent standard deviations of the means of biological replicates.

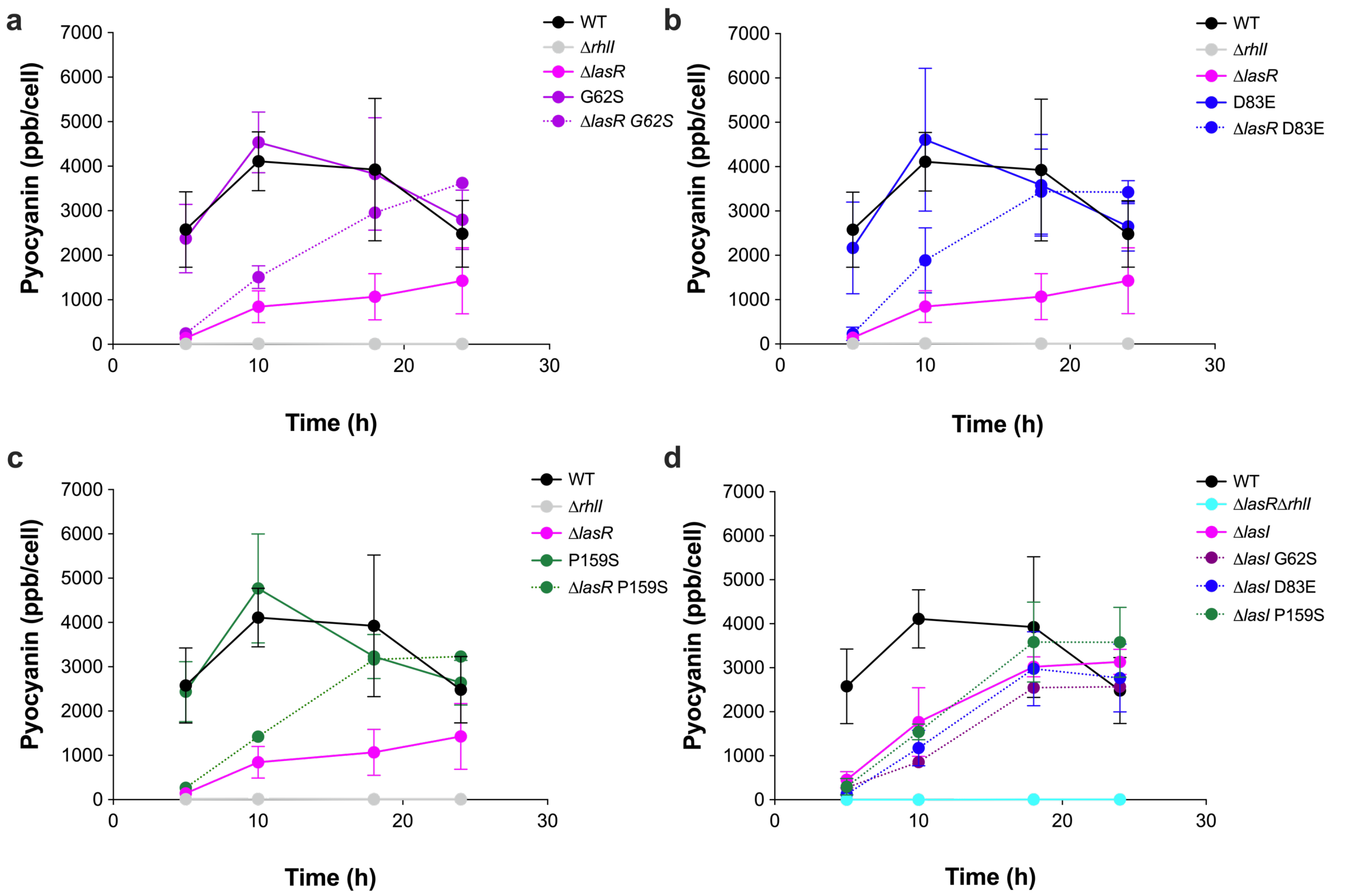

Figure S5: Pyocyanin production in *P. aeruginosa* mutant strains measured by HRMS over time. Absolute pyocyanin levels of PA14 control strains WT (black), Δ*rhlI* (gray) and Δ*lasR* (magenta), as well as the (a) G62S variants, (b) D83E variants, (c) P159S variants in LasR+ (solid lines) and LasR- (Δ*lasR,* dashed lines) backgrounds. (d) Pyocyanin synthesized by RhlI variants in a Δ*lasI* background. All pyocyanin measurements are normalized to the OD_{600} at each time point. Bars represent the mean of three biological replicates. Error bars represent standard deviations of the means of biological replicates.

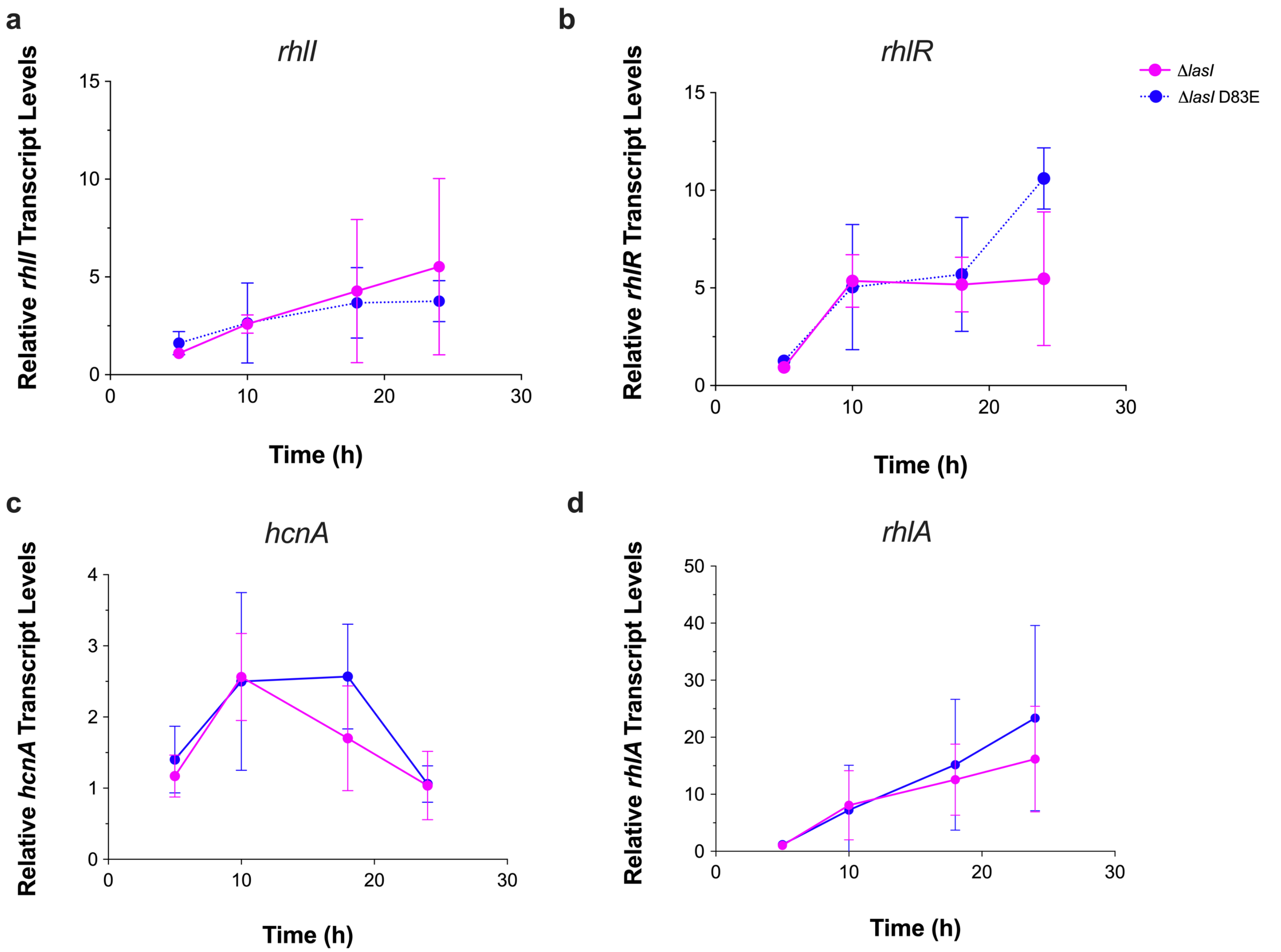

Figure S6: RhlI variants increase *rhlR* transcription in a Δ*lasI* strain over time. The relative transcript levels of (a) *rhlI* (b) *rhlR* (c) *hcnA*, and (d) *rhlA* in Δ*lasI* (magenta) and Δ*lasI* RhlI D83E (blue) strains. Gene expression was normalized to *gyrA* in the Δ*lasI* strain at the 5-hour time point. Bars represent the mean of three biological replicates performed in technical duplicate. Error bars represent standard deviations of the means of biological replicates.

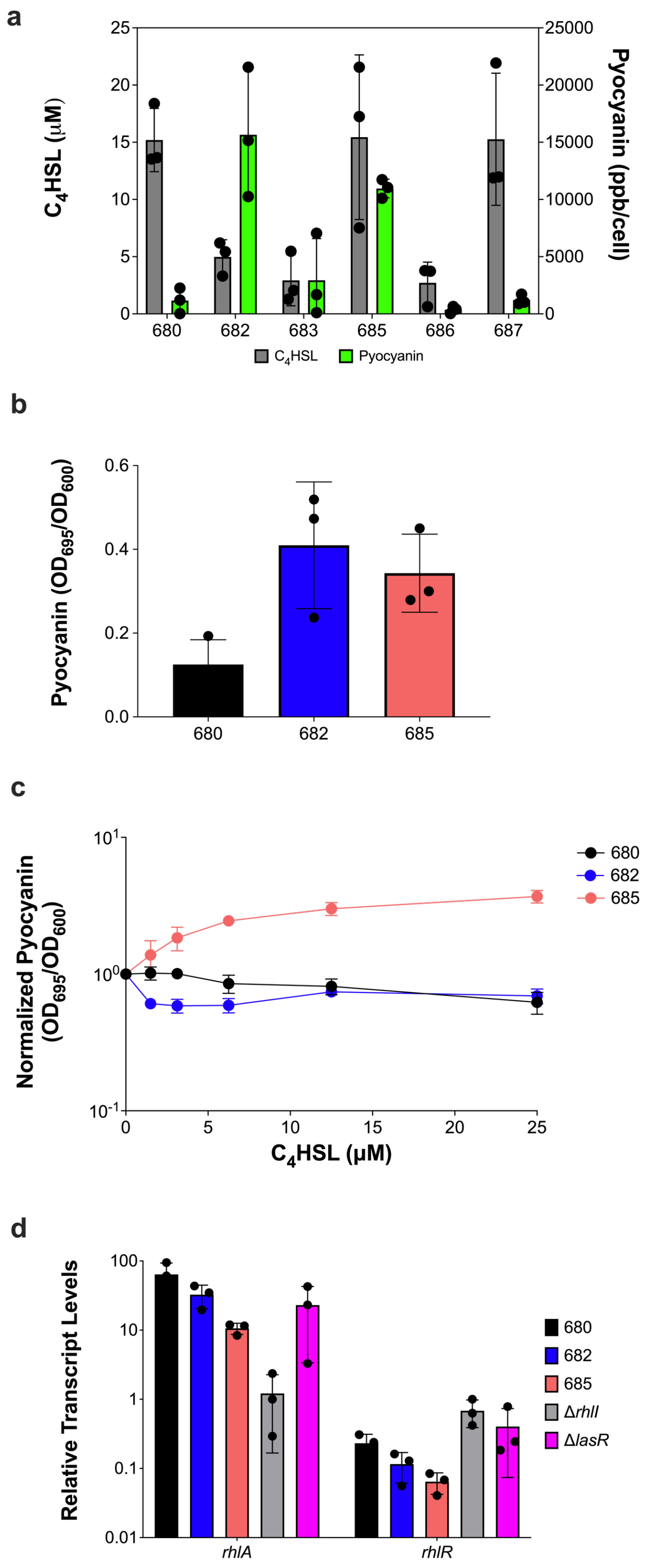

Figure S7: Virulence phenotype expression in clinical isolates is dependent on C_4HSL levels. (a) Pyocyanin and C_4HSL production of select clinical isolates with different *lasR* and *rhlI* mutations measured by HRMS using cell-free supernatant of high-density cultures grown in phosphate-limiting media. (b) Absolute pyocyanin levels using absorbance measurements. (c) C_4HSL dose-response assay of clinical strains grown in phosphate-limiting media. (d) qRT-PCR of *rhlA* and *rhlR* in clinical strains and PA14 Δ*lasR* and Δ*rhlI*. Bars represent the mean of three biological replicates performed in technical duplicate. Error bars represent standard deviations of the means of biological replicates. Gene expression was normalized to *gyrA* for Δ*rhlI.* All experiments were performed in biological triplicate.

II. Supplementary Tables

Table S1. *QS genotypes observed in the 56 clinical isolate cohort.*

SRR number	Source	LasR truncations and deletions	LasR substitutions	RhlI substitutions	Paczkowski lab strain ID
SRR13156973	urine isolate	1-239		D83E	
SRR13156785	wound isolate	1-239		WT	680
SRR13130527	urine clean catch isolate	1-239		D83E	
SRR13085620	urine isolate	1-239	R61S	D83E	
SRR13158623	urine isolate	1-239		D83E	
SRR13158585	knee isolate	1-239		WT	
SRR13156855	urine isolate	1-239		WT	
SRR13130543	tracheal aspirate isolate	1-239		WT	
SRR13130413	tracheal aspirate isolate	1-239		D83E	
SRR13156781	bronchoalveolar lavage isolate	1-172	V171R	D83E	
SRR13130516	blood isolate	1-239		D83E	
SRR13119849	urine isolate	1-239		D83E	
SRR13130514	sputum isolate	1-239		D83E	
SRR13088655	rectal swab	1-151, 153-239		D83E	
SRR13156788	ulcer isolate	1-239		D83E	
SRR13158616	blood isolate	1-239		D83E	
SRR13158527	blood isolate	1-239		D83E, G62S	
SRR13158548	sputum tracheal aspirate isolate	1-87, 116-239		D83E, G62S	
SRR13158481	urine isolate	1-239	L236P	D83E, G62S	683
SRR13158660	leg isolate	1-144, 153-239		D83E, G62S	
SRR13156975	foot isolate	1-148		D83E, G62S	
SRR13158514	blood isolate	116-239		D83E, G62S	682
SRR13156968	urine isolate	1-239		D83E, G62S	
SRR13158501	tracheal aspirate isolate	1-59, 116-239		D83E	
SRR13130517	sputum isolate	1-80, 116-239		D83E	685
SRR13158622	sputum induced isolate	1-239		D83E	686
SRR13156878	sputum isolate	1-239		D83E	

SRR13158638	rectal swab	1-239	G162D	D83E	
SRR13158542	rectal swab	1-239	G162D	D83E	
SRR13158634	tracheal aspirate isolate	1-239	A231V	D83E, P159S	687
SRR13158656	urine isolate	1-239	G191D	D83E, P159S	
SRR13158546	rectal swab	1-239	G191D	D83E	
SRR13158537	abdomen isolate	1-239		D83E	
SRR13145343	urine isolate	1-239	1-4, 9-239	D83E	
SRR13156977	urine isolate	1-172		D83E	
SRR13156852	urine isolate	1-172		D83E	
SRR13158663	urine isolate	1-172		D83E	
SRR13156789	urine isolate	1-172		D83E	
SRR13158497	urine isolate	1-172		D83E	
SRR13156870	urine isolate	1-172		D83E	
SRR13156967	urine isolate	1-172		D83E	
SRR13145342	urine isolate	1-173	K173N	D83E	
SRR13158480	urine isolate	1-172		D83E	
SRR13158505	urine isolate	1-172		D83E	
SRR13156880	urine isolate	1-172		D83E	
SRR13158479	urine isolate	1-172		D83E	
SRR13156871	urine isolate	1-172		D83E	

Table S2. *Strains and plasmids used in this study.*

Strain	Genotype	Plasmid	Resistance	Source
JPS0153	PA14 Δ*lasI*			Mukherjee *et al*. 2017[4]
JPS0154	PA14 Δ*rhlI*			Mukherjee *et al*. 2017[4]
JPS0156	PA14 Δ*lasR*			Mukherjee *et al*. 2017[4]
JPS0222	WT PA14			Gift from George O'Toole
JPS0841	PA14 Δ*lasR rhlI* (D83E)			this study
JPS0842	PA14 *rhlI* (D83E)			this study
JPS0847	PA14 Δ*lasR rhlI* (G62S)			this study
JPS0900	PA14 Δ*lasR* rhlI (P159S)			this study
JPS0901	PA14 *rhlI* (G62S)			this study
JPS0958	PA14 *rhlI* (P159S)			this study
JPS0976	PA14 Δ*lasI* rhlI (G62S)			this study
JPS1013	PA14 Δ*lasR* Δ*rhlI*			this study
JPS1014	PA14 Δ*lasI rhlI* (D83E)			this study
JPS1025	PA14 Δ*lasI rhlI* (P159S)			this study
JPS0737	*E. coli* DH5a	pEXG2-*rhlI*	Gent	this study
JPS0806	*E. coli* DH5a	pEXG2-*rhlI* (G62S)	Gent	this study
JPS0828	*E. coli* DH5a	pEXG2-*rhlI* (P159S)	Gent	this study
JPS0830	*E. coli* DH5a	pEXG2-*rhlI* (D83E)	Gent	this study

Table S3. Primers used in this study.

Name	Sequence	Purpose
oJP1320	ttatt aagctt TTCGAGCGCGAGGAAATCCG	*rhlI* for pEXG2 (HindIII)
oJP1321	ttatt ggatcc AAATCGCGCATCAGGTTCGG	*rhlI* for pEXG2 (BamHI)
oJP1326	CAACACGATATCCAGCCCCT	*hcnA* RT primer
oJP1327	CATTGAGCACGTTGAGCACG	*hcnA* RT primer
oJP1328	CCTGGCCGAACATTTCAACG	*rhlA* RT primer
oJP1329	TTTCCACCTCGTCGTCCTTG	*rhlA* RT primer
oJP1330	GAGGAACTGGAAGCGGTCAA	*gyrA* RT primer
oJP1331	CTTCCTCGGTGATCAGGTCG	*gyrA* RT primer
oJP1414	CATGGCACCTATCCCAAGGC	*rhlR* RT primer
oJP1415	GTCGCTCCAGACCACCATTT	*rhlR* RT primer
oJP1416	CCGAGCTGGGGATGAAGATA	*rhlI* RT primer
oJP1417	CCGTTGCGAACGAAATAGCG	*rhlI* RT primer
oJP1426	gccctggcggctcatggcgacga	*rhlI* Gly62Ser g184a
oJP1427	tcgtcgccatgagccgccagggc	*rhlI* Gly62Ser *g184a*
oJP1428	caggtaggcgaagacctccttgagcaggtag	*rhlI* Asp83Glu *c249a*
oJP1429	ctacctgctcaaggaggtcttcgcctacctg	*rhlI* Asp83Glu *c249a*
oJP1502	cttctgcggcgagccgaggcgct	*rhlI* Pro159Ser *c475t*
oJP1503	agcgcctcggctcgccgcagaag	*rhlI* Pro159Ser *c475t*

III. Supplementary References

1. Bell, E. W. & Zhang, Y. DockRMSD: An open-source tool for atom mapping and RMSD calculation of symmetric molecules through graph isomorphism. *J Cheminform* **11**, 1–9 (2019).
2. Kuznetsov, D. *et al.* OrthoDB v11: annotation of orthologs in the widest sampling of organismal diversity. *Nucleic Acids Res* **51**, D445–D451 (2023).
3. Winsor, G. L. *et al.* Enhanced annotations and features for comparing thousands of Pseudomonas genomes in the Pseudomonas genome database. *Nucleic Acids Res* **44**, D646–D653 (2016).
4. Mukherjee, S., Moustafa, D., Smith, C. D., Goldberg, J. B. & Bassler, B. L. The RhlR quorum-sensing receptor controls Pseudomonas aeruginosa pathogenesis and biofilm development independently of its canonical homoserine lactone autoinducer. *PLoS Pathog* **13**, e1006504 (2017).
