# Supplementary material for: Quorum-sensing synthase mutations re-calibrate autoinducer concentrations in clinical isolates of Pseudomonas aeruginosa to enhance pathogenesis
Source: Nat Commun. 2023 Dec 2;14:7986. doi: 10.1038/s41467-023-43702-4 (PMC10693556; doi:10.1038/s41467-023-43702-4)
Supplement: Supplementary file 3 — Reporting Summary [file 41467_2023_43702_MOESM3_ESM.pdf]

nature portfolio

Corresponding author(s): Jon Paczkowski

Last updated by author(s): 2023/11/07

Reporting Summary

Nature Portfolio wishes to improve the reproducibility of the work that we publish. This form provides structure for consistency and transparency in reporting. For further information on Nature Portfolio policies, see our Editorial Policies and the Editorial Policy Checklist.

Statistics

For all statistical analyses, confirm that the following items are present in the figure legend, table legend, main text, or Methods section.

n/a	Confirmed	
☐	☒	The exact sample size (*n*) for each experimental group/condition, given as a discrete number and unit of measurement
☐	☒	A statement on whether measurements were taken from distinct samples or whether the same sample was measured repeatedly
☐	☒	The statistical test(s) used AND whether they are one- or two-sided<br>*Only common tests should be described solely by name; describe more complex techniques in the Methods section.*
☒	☐	A description of all covariates tested
☐	☒	A description of any assumptions or corrections, such as tests of normality and adjustment for multiple comparisons
☐	☒	A full description of the statistical parameters including central tendency (e.g. means) or other basic estimates (e.g. regression coefficient) AND variation (e.g. standard deviation) or associated estimates of uncertainty (e.g. confidence intervals)
☐	☒	For null hypothesis testing, the test statistic (e.g. *F*, *t*, *r*) with confidence intervals, effect sizes, degrees of freedom and *P* value noted<br>*Give P values as exact values whenever suitable.*
☒	☐	For Bayesian analysis, information on the choice of priors and Markov chain Monte Carlo settings
☒	☐	For hierarchical and complex designs, identification of the appropriate level for tests and full reporting of outcomes
☒	☐	Estimates of effect sizes (e.g. Cohen's *d*, Pearson's *r*), indicating how they were calculated

Our web collection on statistics for biologists contains articles on many of the points above.

Software and code

Policy information about availability of computer code

Data collection	Mass spectrometru data were acquired with Xcalibur 4.1 and processed with TraceFinder 5.0, both from ThermoFisher.
Data analysis	Whole genome assembly and phylogenetic analyses<br>Paired-end reads were downloaded from the National Center for Biotechnology Information BioProject database (NCBI accession number: PRJNA288601). Reads were trimmed and assessed for quality using Trim Galore! v0.6.750,51. Trimmed reads were mapped to the P. aeruginosa UCBPP-PA14 reference assembly (NCBI accession number: NC_008463.1) using the BWA-MEM 52 algorithm within SAMtools v1.1.053. Variant calling and filtering were performed by BCFtools mpileup v1.1.0.254. Consensus FASTA sequences were generated using BCFtools consensus V1.1.0.2. To identify RhlI SNPs and sequence relatedness, consensus sequences were annotated using Prokka v1.1455, using default parameters. RhlI-annotated protein sequence alignments were generated using MUSCLE v3.8.155156. Columns with >20% gaps were trimmed and filtered by similarity with trimAl (v1.4.1; option --gt 0.80 and --st 0.001)57. The maximum-likelihood tree was generated from RhlI protein sequence alignments (n=56) with IQ-TREE v1.6.1258. Model selection was performed using an automatic substitution model based on the Bayesian information criteria (BIC) score, where the HKY+F+I model was chosen. The tree was visualized and annotated with Interactive Tree Of Life (iTOL v. 6.7.1)59. To identify RhlI variants among the isolates, we used the Pseudomonas Genome DB60. We utilized the NCBI BLAST61,62 search function to compare rhlI isolate sequences to the P. aeruginosa UCBPP-PA14 reference assembly (NCBI accession number: NC_008463.1). Mismatches were identified using the pairwise output format.<br>To contextualize RhlI sequence similarity among a diverse range of bacterial species, we obtained protein sequences of RhlI orthologs from the OrthoDB v1163 online database. Protein sequences were filtered for those which were between 185-215 amino acids. The remaining sequences were aligned using MUSCLE v3.8.1551 and trimmed with trimAl v1.4.1. The maximum-likelihood tree was generated from orthologous RhlI protein sequence alignments (n=264) with IQ-TREE v1.6.12. Model selection was performed using an automatic substitution model based on the Bayesian information criteria (BIC) score, where the LG+F+R7 model was chosen. The tree was visualized and annotated

with Interactive Tree Of Life (iTOL) v6.7.1. Custom code and all commands issued in this study can be found at https://github.com/calebmallery/RhlI_manuscript_methods.
Statistical analyses and graphical depictions were performed with GraphPad Prism 9.5.1

For manuscripts utilizing custom algorithms or software that are central to the research but not yet described in published literature, software must be made available to editors and reviewers. We strongly encourage code deposition in a community repository (e.g. GitHub). See the Nature Portfolio guidelines for submitting code & software for further information.

Data

Policy information about availability of data

All manuscripts must include a data availability statement. This statement should provide the following information, where applicable:

- Accession codes, unique identifiers, or web links for publicly available datasets
- A description of any restrictions on data availability
- For clinical datasets or third party data, please ensure that the statement adheres to our policy

Genome assembly for strains used in this study were previously deposited to the National Center for Biotechnology Information BioProject # PRJNA288601 and are

Research involving human participants, their data, or biological material

Policy information about studies with human participants or human data. See also policy information about sex, gender (identity/presentation), and sexual orientation and race, ethnicity and racism.

Reporting on sex and gender	N/A
Reporting on race, ethnicity, or other socially relevant groupings	N/A
Population characteristics	N/A
Recruitment	N/A
Ethics oversight	N/A

Note that full information on the approval of the study protocol must also be provided in the manuscript.

Field-specific reporting

Please select the one below that is the best fit for your research. If you are not sure, read the appropriate sections before making your selection.

☒ Life sciences ☐ Behavioural & social sciences ☐ Ecological, evolutionary & environmental sciences

For a reference copy of the document with all sections, see nature.com/documents/nr-reporting-summary-flat.pdf

Life sciences study design

All studies must disclose on these points even when the disclosure is negative.

Sample size	All experiments were performed in biological triplicate with technical duplicates per experiment unless stated otherwise. Sample size was chosen based on the assay and previous studies that conducted similar studies in the field of the microbiology. We followed established protocols for all cell-based assays.
Data exclusions	No data were excluded in the course of this study.
Replication	Experimental results were reproducible across all biological samples. Each experiment was performed independently at least three times on different days with different starter cultures to ensure validity and reproducibility.
Randomization	Our experiments did not require randomization as each strain of bacteria was its own experimental group.

Blinding | Strain identifiers were blinded to the investigators at the outset of the study.

Reporting for specific materials, systems and methods

We require information from authors about some types of materials, experimental systems and methods used in many studies. Here, indicate whether each material, system or method listed is relevant to your study. If you are not sure if a list item applies to your research, read the appropriate section before selecting a response.

Materials & experimental systems

n/a	Involved in the study
☒	☐ Antibodies
☒	☐ Eukaryotic cell lines
☒	☐ Palaeontology and archaeology
☒	☐ Animals and other organisms
☒	☐ Clinical data
☒	☐ Dual use research of concern
☒	☐ Plants

Methods

n/a	Involved in the study
☒	☐ ChIP-seq
☒	☐ Flow cytometry
☒	☐ MRI-based neuroimaging

Plants

Seed stocks | N/A

Novel plant genotypes | N/A

Authentication | N/A
